# Modelling actin polymerization: the effect on confined cell migration

**DOI:** 10.1007/s10237-019-01136-2

**Published:** 2019-03-01

**Authors:** S. Hervas-Raluy, J. M. Garcia-Aznar, M. J. Gomez-Benito

**Affiliations:** Universidad de Zaragoza, Campus Rio Ebro, 50018 Zaragoza, Spain

**Keywords:** Cell migration, Confined migration, Finite elements, Mechanical modelling, Cancer metastasis

## Abstract

The aim of this work is to model cell motility under conditions of mechanical confinement. This cell migration mode may occur in extravasation of tumour and neutrophil-like cells. Cell migration is the result of the complex action of different forces exerted by the interplay between myosin contractility forces and actin processes. Here, we propose and implement a finite element model of the confined migration of a single cell. In this model, we consider the effects of actin and myosin in cell motility. Both filament and globular actin are modelled. We model the cell considering cytoplasm and nucleus with different mechanical properties. The migration speed in the simulation is around 0.1 μm/min, which is in agreement with existing literature. From our simulation, we observe that the nucleus size has an important role in cell migration inside the channel. In the simulation the cell moves further when the nucleus is smaller. However, this speed is less sensitive to nucleus stiffness. The results show that the cell displacement is lower when the nucleus is stiffer. The degree of adhesion between the channel walls and the cell is also very important in confined migration. We observe an increment of cell velocity when the friction coefficient is higher.

## Introduction

Interstitial cell migration plays an important role in numerous cellular processes such as tissue formation and regeneration, immune cell trafficking, and disease, including cancer invasion and metastasis. In confined cell migration, cells move through confined spaces, and both the cytoplasm and nucleus must be deformed to pass through the available spaces. In the inflammatory response, leukocytes circulate in the bloodstream, and as they enter an area of inflammation, they attach to the endothelium, pass through it and migrate through tissues to reach the site of infection (Friedl and Weigelin 2008). In metastasis, tumour cells migrate from the initial tumour mass to the circulatory system, which they later leave and migrate to a new location. Cancer cells show high deformation capacity, and it allows them to circulate along tortuous and confined environments and to get out the vessel (Leber and Efferth 2009). In fact, cancer cells and leukocytes use similar strategies to spread throughout the body (Madsen and Sahai 2010).

Both cancer cells and leukocytes share common features during their migration. To extravasate, cancer cells and leukocytes must strongly change their shape. In these cases, cell motility results from the combination of actin polymerization in the leading edge and myosin contraction at the rear part of the cell. First, actin polymerization begins with globular actin (G-actin). G-actin is a monomer which is located initially surrounding the nucleus. Then, G-actin moves towards the cell front; when there is a high concentration of it at the leading edge, it will polymerize converting into filament actin (F-actin) (Alberts et al. 2005). The elongated actin filaments push the membrane forward generating protrusions (Mogilner et al. 2003). Second, myosin located in the cell rear exerts contraction forces. Actin–myosin network is responsible for force generation in smooth and striated muscles as well as in non-muscle cells during cellular motility. The contraction exerted by myosin at the rear is as important as the polymerization at the leading edge for cell movement. Additionally, cell adhesion to the substrate is also necessary to promote this kind of migration.

In this field, Wilson et al. (2013) have made a great deal of progress studying the leading edge protrusion. They have used a microfluidic device to study the front part of the cell during confined migration. Their results prove that there are two different actin networks, one at the cell front (free F-actin network) where polymerization is faster and oriented in the direction of motion and another located at the cell–wall interface (adherent F-actin network) where polymerization is slower and directed perpendicular to the channel wall. In addition, these two actin networks can interact mechanically: the growth in the middle of the adhered F-actin network might compress the free F-actin network, preventing its retrograde flow and allowing new polymerization at the free membrane to generate protrusion.

Regarding the rear part of the cell, Chabaud et al. (2015) have studied where myosin locates during cell migration. They introduced leukocytes into a microfluidic device and observed that while migrating, myosin is located at the rear part of the cell; however, when the cell stops, myosin distributes both at the front and the rear of the cell. In addition, myosin distribution is not uniform in the migration process: there is a peak at the rear, and there is less concentration near the nucleus. In fact, myosin concentration depends on the distance to the nucleus.

Therefore, actin polymerization and myosin contraction per se are not enough to promote cell migration. The coordinated movement depends on the development and maintenance of functional asymmetry, mostly known as polarization (Verkhovsky et al. 1999). Therefore, cells exhibit a morphological polarization, with the front and rear being easily distinguishable. This polarization may be initiated by protrusion at the leading edge (Weiner et al. 1999) or by retraction at the rear part of the cell (Verkhovsky et al. 1999). However, it has not been determined how polarization initiates and propagates in the absence of an external stimulus (Yam et al. 2007). In addition, when the cell migrates inside a confined channel, the cytoskeleton (CSK) adapts to the channel shape, and most actin filaments align in the direction of the movement (Paul et al. 2016).

Cell migration in confined environments has been studied in many different works, both in vitro and in silico. Regarding in vitro experiments, the usual picture of cell locomotion is then as follows: the cell lamellipodium builds strong adhesion points with the substrate and pushes forward its membrane by actin polymerization. At the back, the cell body contracts and breaks the adhesion points (Pollard and Borisy 2003; Le Clainche and Carlier 2008; Gao and Gao 2016). Different authors (Hawkins et al. 2009; Lämmermann et al. 2008) have suggested a simple mechanism which is mainly powered by actin polymerization at the cell membrane and strongly relies on geometry confinement. Moreover, these models do not need strong specific adhesion. So, if adhesion is an important factor in confined migration is still unclear. In fact, Heuze et al. (2013) have pointed out that cells migrating in two-dimensional substrates form adhesions with the extracellular matrix, and those who migrate in three-dimensional mode are exerting pressure forces against the channel wall, generating high friction which will be the key of this kind of movement (Hawkins et al. 2009). However, the principles under cell confined motility remain not completely understood.

In silico models have been shown to be an important tool to understand the mechanics of cell migration. Different computational models have been proposed; they can be classified according to several factors such as the geometrical configuration: one dimension (1D) (Recho et al. 2014; Mogilner et al. 2001), two dimensions (2D) (Chen et al. 2018; Rubinstein et al. 2005) or three dimensions (3D) (Moure and Gomez 2017; Kim et al. 2015; Merino-Casallo et al. 2018). In addition, they can also be classified according to the cell scale as subcellular models, which explain particular processes such as the role of actin–myosin network (Borau et al. 2012) or simulating the entire cell in cellular-scale models (Moure and Gomez 2016). There are also models that simulate entire cell clusters (Escribano et al. 2018).

Furthermore, there are different ways to approach the physical problem to be modelled. Rubinstein et al. (2005) have proposed a multiscale 2D model which includes actin protrusion in the front part of the cell, actin–myosin contraction at the rear and actin transport across the whole cell. Recho et al. (2013) have presented a mathematical fluid model developed in 1D, which remarks the crucial role of myosin contraction during migration. The model is based on the symmetry-breaking instability of a non-motile configuration and ensuring directional motility is enough to make the cell migrate.

Recent models place more emphasis on modelling confined cell migration. Moure and Gomez (2018) have presented a phase-field model of the spontaneous migration of a single cell. Their model is based on a cell domain delimited by a membrane-bound activator. In this model, myosin is transported by the actin network and diffuses throughout the cell. Actin is differentiated into G-actin and F-actin, expressed by a bistable equation. They have used the phase-field method to adapt to the large strain of the cell while migrating, and they have assumed the cell as a viscous fluid. In parallel, Chen et al. (2018) have developed a phenomenological model which simulates the deformation of the cell and the nucleus during invasion through a dense, physiological environment. In their work, they have simulated the CSK as a collection of springs, and they have also include chemotactic movement. They have modelled both 2D and 3D cells inside confined environments, and the obstacles were assumed as rigid solids. They have used an implicit–explicit integration method where the linear parts and the nonlinear parts are treated using an Euler backward scheme and an Euler forward method, respectively. In both models, evolution of F-actin and G-actin is analysed together with cell movement.

Computational models have proved the important role of nucleus deformability (Serrano-Alcalde et al. 2017). Cao et al. (2016) have investigated the impact of the confined migration on the geometrical and mechanical features of cell nucleus. Some authors (Aubry et al. 2015; Giverso et al. 2014; Scianna and Preziosi 2013) have simulated the cell nucleus separately during the migration process in microfluidics, highlighting the key role of the nucleus deformability in cell migration. Therefore, cell nucleus presents a key role in confined cell migration, and it is the limiting component since it is much stiffer than the cell cytoplasm.

The aim of our work is to develop a model of cell migration which takes into account the different dynamics of cytoskeletal structures: myosin, G-actin and F-actin. The final aim is to study cell migration from a mechanical point of view to investigate possible mechanism of mechanotransduction inside the cell.

## Materials and methods

### An actin-based mechanical model for confined cell migration

We present a new model for cell motility in confined environments. In this model, we consider the effects of actin and myosin in cell motility. Both F-actin and G-actin are modelled.

At the cell leading edge, actin polymerization occurs rapidly due to the high concentration of globular actin and the presence of actin filaments, which act as a nucleus for filament growth.

The actin filaments’ ends orientation makes the two ends of each polymer different in ways that have a profound effect on filament growth rates. The kinetic rate constants for actin subunit polymerization ($K_{{\rm ON}}$) and depolymerization ($K_{{\rm OFF}}$) are much greater at the ends (Alberts et al. 2005).

G-actin is presented as a monomer, and when it polymerizes, it converts in F-actin. G-actin is mainly located at the leading edge, in order to polymerize into F-actin. F-actin near the nucleus starts to depolymerize, becoming G-actin again. So there is an important reservoir of G-actin near the nucleus, which tends to move towards the front, so it can form F-actin and promote cell migration.

The experimental data revealed (Wilson et al. 2013) that actin polymerization takes place predominantly in two locations. First, at the leading edge, actin polymerizes at the free membrane (free F-actin network) where polymerization is stronger and it is oriented in the direction of motion. Second, at the cell–wall interface (adherent F-actin network) polymerization is directed outwardly, perpendicular to the channel wall and it contributes to ensure the cell–channel contact. This polymerization is high enough to maintain the contact pressure, but not so large to deform the nucleus.

Equation 1 quantifies the net change rate of F-actin concentration over time (*t*). F-actin is responsible for actin polymerization, which takes place where there is a high concentration of G-actin, and if F-actin is near the cell membrane. As well, depolymerization mainly takes place near the nucleus if there is a high concentration of F-actin. Thus, the net rate change of F-actin concentration depends also on the concentration of the G-actin (Mogilner and Edelstein-Keshet 2002) and the distance to the cell front:

1 $$\frac{\partial \rho _f({\mathbf{x}},t)}{\partial t} = K_{{\rm ON}}\frac{\rho _{g}^2}{\rho _g^2+\lambda }\rho _f({\mathbf{x}},t)\delta _f({\mathbf{x}})- K_{{\rm OFF}}\rho _f({\mathbf{x}},t)\delta _c({\mathbf{x}})$$

where $\rho _{g}$ is G-actin concentration, $\rho _f$ is F-actin concentration, $\delta_ i({\mathbf{x}})$ is a function that depends on the position, $\lambda$ is a constant and **x** are the coordinates of the point at time *t*.

Free actin polymerizes at the leading edge, and it makes the cell moves forward and depolymerizes near the nucleus. The net range of F-actin change is not equal in the whole cell; it depends on the longitudinal coordinate (Rubinstein et al. 2005), as it has been observed in experimental data (Wilson et al. 2013), so the function $\delta_i$ must be defined:

2 $$\delta _i({\mathbf{x}}) ={\mathscr{H}} (d_{i0}^{{\rm ef}} - d_{i0}({\mathbf{x}}))$$

where ${\mathscr{H}}$ is the Heavyside function, and index *i* can be *c*, centre, or *f*, front. Then, $d_{c0}^{{\rm ef}}$ is equal to the effective distance where depolymerization takes place (3 μm to the nucleus (Mogilner and Edelstein-Keshet 2002)) and $d_{f0}^{{\rm ef}}$ is the effective distance where polymerization is occurring, [2 μm to the cell front (Wilson et al. 2013)]. $d_{c0}({\mathbf{x}})$ is the distance between the point **x** and the cell nucleus, and $d_{f0}({\mathbf{x}})$ is the distance between the point **x** and the cell front.

Note that actin is polymerizing mainly at the cell front and depending on the G-actin concentration. The relationship between these two variables is not linear; only when there is a high concentration of G-actin, polymerization occurs (Rubinstein et al. 2005). The last term of the equation represents depolymerization, it is negative because depolymerization transforms F-actin into G-actin, and it mostly happens near the nucleus.

Equation 3 defines the net change rate of G-actin concentration, which represents the depolymerization process. As actin polymerization is taking place at the front of the cell, actin depolymerization takes place close to the nucleus. When depolymerization occurs, F-actin transforms into G-actin. G-actin can move inside the cell; this movement is assumed to follow a random walk model which could be modelled by Fick’s law. Thus, the net rate change of G-actin concentration follows:

3 $$\begin{aligned} \frac{\partial \rho _g({\mathbf{x}},t)}{\partial t} &=\bigtriangledown ({\mathbf{D}} \bigtriangledown \rho _g({\mathbf{x}},t)) \\ &\quad-\, K_{{\rm ON}}\frac{\rho _{g}^2}{\rho _g^2+\lambda }\rho _f({\mathbf{x}},t)\delta ({\mathbf{x}}) \\ &\quad+\, K_{{\rm OFF}}\rho _f({\mathbf{x}},t)\delta ({\mathbf{x}}) \end{aligned}$$

where **D** is the diffusivity tensor.

First, when F-actin depolymerizes, G-actin is generated at the front part of the cell, close to the nucleus. Then, this G-actin moves forward to the cell front, ready to polymerize with actin filaments.

The G-actin diffusion coefficient is maximum between the nucleus and the front, and it is zero in the cell rear. G-actin spreads by diffusion from the filament’s pointed ends towards the barbed ends (Novak et al. 2008).

Actin is continuously polymerizing and depolymerizing; thus, it transforms from F-actin to G-actin and also in the backwards direction. However, the total amount of actin remains constant (*c*) inside the whole cell while migrating, and thus:

4 $$\int _{\varOmega }^{}(\rho _f+\rho _g){\rm d}\varOmega = c$$

In addition, myosin contraction has an important role in cell migration. Myosin is a molecular motor that provokes cellular contraction, and it is responsible for the cell rear contraction, contributing to cell migration. Myosin is not evenly distributed; we assume that the contraction level depends on the longitudinal coordinate following experimental observations (Chabaud et al. 2015). We neglect the net change in myosin with time; thus, myosin is assumed to be constant throughout the analysis; however, it can be attached or detached to the CSK. Equation 5 quantifies the myosin concentration, which is maximum close to the cell rear and it decreases depending on the distance to the nucleus.

5 $$\rho _m = k \delta _m({\mathbf{x}})$$

where $\rho _m$ is the concentration of myosin, *k* is a constant and $\delta _m({\mathbf{x}})$ is the distance between the point **x** and the nucleus.

To simulate cell polymerization, depolymerization and myosin contraction, we assume that these proteins produce volumetric cell contraction or expansion. We make use of the multiplicative decomposition (Vujosevic and Lubarda 2002) of the total deformation gradient **F**:

6 $${\mathbf{F}} = {\mathbf{F}}_e \cdot {\mathbf{F}}_a \cdot {\mathbf{F}}_m$$

where ${\mathbf{F}}_e$ is the isothermal deformation gradient, ${\mathbf{F}}_a$ is the deformation gradient produced by the volume change due to polymerization (expansion) and depolymerization (contraction), which is defined as:

7 $${\mathbf{F}}_{a} = \alpha _f \frac{\partial \rho _f({\mathbf{x}},t)}{\partial t} {\mathbf{e}}_{1}\otimes {\mathbf{e}}_{1}$$

where $\alpha _f$ is a constant and ${\mathbf{e}}_{1}$ is the preferential polymerization direction.

${\mathbf{F}}_m$ is the deformation gradient due to myosin contraction, which is assumed to be proportional to the concentration of myosin and it provokes a volume reduction:

8 $${\mathbf{F}}_{m} = \alpha _m \rho _m{\mathbf{1}}$$

where $\alpha _m$ is a constant.

The nucleus plays an important role in distributing the protein concentration. Petrie et al. (2014) show that in cells migrating into confined 3D spaces the nucleus physically divides the cytoplasm into front and tail. Therefore, the distance from an x-point to the nucleus will serve to determine which protein is involved.

In 3D confined cell migration, several experiments prove that the compressed cell exerts forces perpendicular to the channel walls (Malawista et al. 2000). They state that friction between the cell and the substrate is enough to promote cell migration. Therefore, in our model cell adhesion is represented by the friction between the cell and the channel (Moure and Gomez 2017).

## Numerical implementation

We simulate the migration of an individual cell inside a confined channel. The cell is confined in the middle of the channel, and it migrates thanks to the actin polymerization and the myosin contraction, following the proposed model. This migration is a consequence of the dynamical interaction of actin polymerization, depolymerization and myosin contraction.

**Fig. 1:**
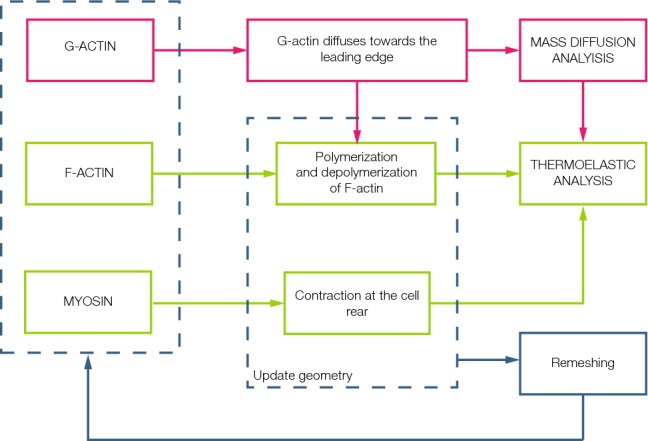
Flowchart of the interactive computational implementation. G-actin, F-actin and myosin are the proteins modelled. A mass diffusion analysis is performed in order to simulate the movement of the G-actin from the nucleus towards the leading edge. The new concentration is an input to the thermoelastic analysis, where the polymerization and depolymerization of the F-actin and the contraction of myosin at the rear are simulated. In this step, cell migration is achieved and the mesh undergo high deformations. Thus, a remeshing code is applied and the new concentrations of the proteins are the initial conditions of the next step. This loop is repeated until the end of the analysis

The finite element method (FEM) is the numerical tool used for development of the simulations (Fig. 1). Two different analyses are performed in order to determinate the evolution of the cell during migration. In the first one, a mass diffusion analysis is performed to simulate the movement of G-actin inside the cell. The second analysis simulates actin polymerization and depolymerization, as well as myosin contraction. This results in a new cell geometry defined through the displacements obtained in this analysis.

In the mass diffusion analysis, G-actin movement is assumed to be random. First, when F-actin depolymerizes, G-actin is generated close to the nucleus. This G-actin moves forward to the cell front, ready to polymerize with F-actin. This process is simulated via Abaqus mass diffusion analysis. Since G-actin diffuses mainly towards the cell front, the diffusion coefficient is orthotropic, following the direction of actin microfilaments, and it is maximum between the nucleus and the front, and it is zero at the cell rear (Novak et al. 2008).

After the mass diffusion analysis, F-actin polymerization takes place. G-actin located at the leading edge joins to the existing actin filaments, forming new F-actin.

Since actin polymerization and depolymerization process involves volume change, this can be approached within a thermoelastic analysis, so the net rate of F-actin concentration is simulated as a temperature gradient, in order to represent the protrusion. In the same way, myosin contraction at the rear is modelled as a negative temperature gradient. Globular actin computed in the diffusion analysis and the F-actin from the previous step are inputs in this analysis. In addition, the adherent F-actin is located in the cell–wall interface, with a 0.5 μm of thickness (Wilson et al. 2013). The polymerization occurs mainly in the longitudinal direction on the free F-actin network; however, the adherent F-actin polymerization occurs in the normal direction to the cell–channel interface. The depolymerization process has not a principal direction. The new cell geometry is defined through the displacements obtained in the thermoelastic analysis.

After these two analyses, the actin concentration is uploaded. With this, the loop is completed and a new time increment consisting in the two analyses is performed again. While migrating, cells are strongly deformed and this results in mesh distortion. To improve the finite element mesh, we perform a remeshing analysis. The deformed cell contour is taken in order to create the new geometry and mesh. The remeshing rule is written in Python language and executed via Abaqus scripts. The aim of this part of the analysis is to replace the distorted mesh with a new one. The remeshing code modifies the number of nodes in each iteration; for example, in the initial step, the number of nodes is 5584 and at the end, this number is 5796. G-actin, F-actin and myosin concentration are extrapolated from the old mesh to the new one. Thus, we adopt an updated Lagrangian formulation. Once we apply the remeshing rule, the iteration is finished, and the next one starts again with the diffusion analysis in the new geometry.

The results are post-processed in Paraview (Ayachit 2015), in order to visualize all the results together. The variables shown in the post-processing are displacements and F-actin, G-actin and myosin concentration.

### FEM simulation

#### Geometry

The model geometry is assumed to be axisymmetric. The cell is modelled as a solid cylinder, distinguishing between cytoplasm and nucleus, and the channel is a hollow cylinder.

**Fig. 2:**
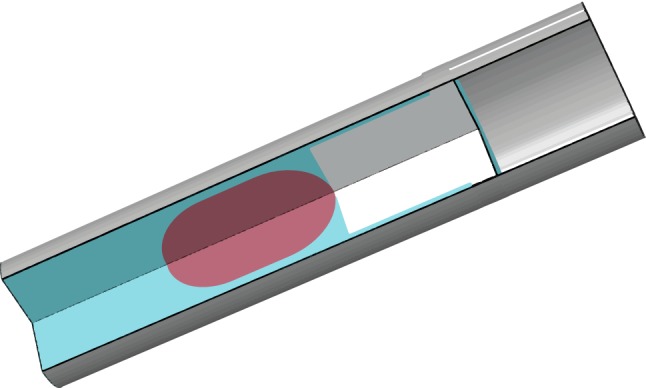
The cell is modelled using three different materials. The nucleus is ten times stiffer than the cytoplasm material. Two different materials can be distinguished in the cytoplasm, where the mechanical properties are the same but the difference lies in the way they change its volume. In the leading edge, the expansion and contraction are orthotropic, and the highest value is oriented in the longitudinal direction. Thus, the polymerization of the free F-actin network is modelled. Elsewhere in the cell, the expansion and contraction are isotropic

The model is composed of two different parts, the channel and the cell (Fig. 2). The channel radius is 6 μm, and its length is long enough to prevent the cell to be out of the channel during simulation.

The cell is divided into nucleus and cytoplasm. The nucleus is simulated as a differentiated part of the cell with different mechanical properties that only participates in the mechanical behaviour of the model. The nucleus is located in the centre of the cell in the initial configuration. The dimensions of the cell are based on the experiments of Bergert et al. (2015). Thus, the cell and nucleus have a length of 40 μm and 15 μm, respectively, in the longitudinal coordinate.

#### Materials

Cell is modelled as a linear elastic material. Two different parts are considered, the cytoplasm (Young modulus, 0.8 kPa; Poisson’s coefficient, 0.38) and the nucleus, which is considered ten times stiffer than the cytoplasm (Young modulus, 8 kPa; Poisson’s coefficient, 0.38) (Friedl et al. 2011; Vaziri et al. 2006; Trepat et al. 2008).

The cell is considered isotropic. However, in order to simulate the orthotropic polymerization of F-actin, the expansion coefficient is the highest in the longitudinal direction.

The channel is modelled as a rigid solid since it is much stiffer than the cell. For this simulation, the parameters $K_{{\rm ON}}$, the rate of polymerization, and $K_{{\rm OFF}}$, the rate of depolymerization are fixed in 0.5 monomers/s (Moure and Gomez 2017). The diffusion coefficient of the globular actin is set as 0.5 μm$^2$/s. The constants are set as: $\lambda = 4$, $\alpha _f=0.5$, *k* = 0.04 and $\alpha _m=0.05$.

We discretize the cell with linear triangular elements. A mesh sensitivity study is performed. As a result, the element side size is 0.25 μm.

#### Initial and boundary conditions

We ensure the initial position of the cell. The cell is confined inside a channel and thus initially pressurized inside it. So the first steps of the simulation are performed to achieve the contact between the cell and the channel wall. The cell size is initially modelled bigger than the channel size. In the first step, a uniform pressure is applied all over the cell surface in order to decrease its size. Then, the cell enters into the channel so it is not in contact with the cell walls. To achieve the original configuration observed in the literature (Wilson et al. 2013; Irimia et al. 2007), the pressure is released in the second step. This step is designed just to allow cell stability inside the channel; the cell will expand and adjust to the channel. Thus, in this configuration the cell is initially pressurized.

Once the cell has raised the “undeformed” configuration, the next steps are the ones which simulate cell migration. The contact between the cell and the channel is modelled as a tangential behaviour. The friction coefficient is dimensionless, and it is varied between 0 and 1; we start with a friction coefficient of 0.5 for the control case. Two more analyses with different friction coefficients (0.4 and 0.6) are developed in order to study the influence of this factor.

As initial conditions, we assume a high concentration of G-actin near the nucleus. In the actin polymerization analysis, the initial conditions are the adherent F-actin concentration. Myosin, which is assumed to be dependent on the distance towards the nucleus, is located in the rear part of the cell.

## Results

In this section, the ability of our model to reproduce the confined cell migration is shown. Our model reproduces G-actin diffusion, as well F-actin polymerization and myosin contraction. We also investigate the influence of the nucleus size and the nucleus stiffness in cell migration. Moreover, the influence of the friction between the cell and the channel wall is analysed.

**Fig. 3:**
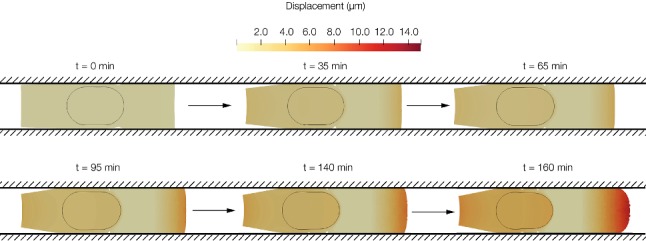
Cell displacement in the longitudinal direction over different times of the simulation. The maximum displacement is 15 μm, and it is measured in the leading edge. At the beginning, the cell is polarizing so the displacement is minor. Once the cell is polarized, the nucleus starts to move towards the leading edge, and greater displacement is obtained

Cell displacement in the longitudinal direction represents cell migration. In Fig. 3 we can see the motility of the cell confined in the channel. The leading edge displaces 15 μm, and it represents the protrusion formation. The average velocity achieved by the cell is 0.1 μm/min, which is similar to the one observed in in vitro experiments (Liu et al. 2015; Irimia and Toner 2009).

**Fig. 4:**
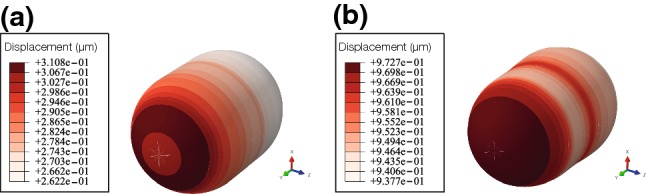
Incremental displacement of the nucleus in two times of the simulation. **a** Displacement between 0 and 10 minutes (mins). **b** Displacement between 140 and 150 mins. At the beginning of the simulation, the cell is polarizing itself so the displacement is low. At the end, the cell is polarized; thus, the displacement is higher

**Fig. 5:**
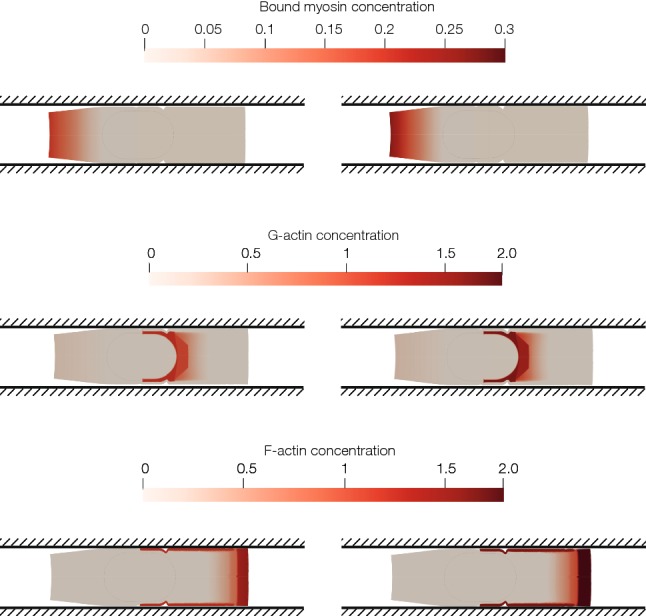
Spatiotemporal proteins’ concentration in time 30 min and 50 min. **a** Concentration of myosin. Myosin is located at the rear part of the cell. Myosin’s concentration depends on the longitudinal coordinate; thus, the lowest value is located near the nucleus and in the front part of the cell. Since the main contraction is generated by myosin attached to the CSK in the rear part of the cell, only this one is modelled. **b** Concentration of G-actin. Depolymerization takes place near the nucleus. Thus, the highest value of the G-actin concentration is located close to the nucleus. **c** Concentration of F-actin. The adherent network of F-actin is located in the cell–wall interface. In the cell front, there is a peak of concentration close to the membrane. Concentration depends on the longitudinal distance to the cell nucleus

Once the cell achieves the characteristic deformed configuration of a cell migrating in a confined channel (the typical tail in the rear of the cell and the nucleus located towards the tail), the displacement is almost the same in the nucleus and in the cell rear. In Fig. 4 we can observe how the displacement of the nucleus at the beginning of the simulation is almost zero compared to the displacement at the end of the simulation. In Fig. 5 the concentration of different proteins can be seen in two different times of the simulation.

**Fig. 6:**
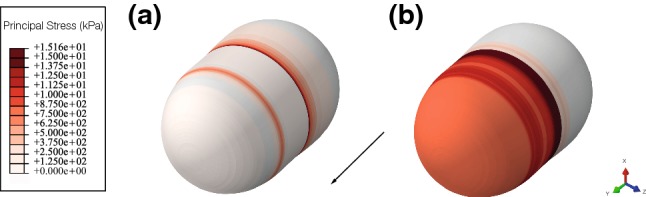
Absolute principal stress (kPa) in the nucleus at the end of the simulation. **a** Absolute maximum principal, **b** absolute minimum principal. The front part of the nucleus is compressing due to the depolymerization process and also because of the compressing forces that the adherent F-actin network is exerting. The rear part of the nucleus is relaxed

Attending to the mechanics of the cell, the nucleus is stiffer than the cytoplasm, so the cytoplasm is deforming more than the nucleus. Mainly, the nucleus is suffering traction stresses, which are mostly zero in the rear part of the nucleus, whereas at the front part are about 0.1 kPa (Fig. 6).

Hereunder, we investigate how different factors affect cell migration. We focus on nucleus size, on nucleus stiffness and friction between the channel walls and the cell.

**Fig. 7:**
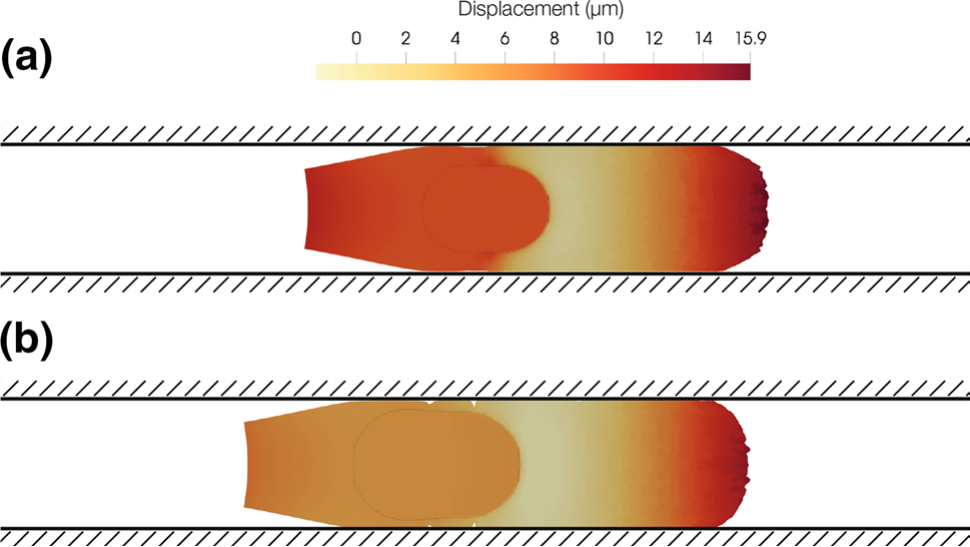
Longitudinal displacement (μm). **a** Cell with a smaller nucleus. **b** Control case. The cell with a smaller nucleus is moving further than the control one. The cell with a smaller nucleus is moving 16 μm, and the control one is moving 15 μm

First, we model a new geometry in which the nucleus size is smaller. The results (Fig. 7) show that cell displacement is actually larger when the nucleus is smaller. The final displacement of the cell is 16 μm, which is bigger than the displacement of the control case, 15 μm.

Secondly, the influence of nucleus stiffness is studied. We simulate three different nucleus with stiffness of 4 kPa, 8 kPa (control case) and 16 kPa. The results show that a lower nucleus stiffness apparently only has a minimal influence during migration. Nevertheless, a higher nucleus stiffness has a strong effect on cell displacement (Table 1).

The maximum cell displacement is nearly the same in the two cases with lower stiffness; however, the cell with the lowest Young modulus presents the greater displacement. The displacement when the nucleus stiffness is 16 kPa is the minor. The nucleus maximum displacement follows the same line, obtaining the maximum when the nucleus stiffness is the lowest.

**Table 1:** Cell displacement depending on the nucleus stiffness. The cell displaces further when the stiffness is the lowest

	4 kPa	8 kPa (control case)	16 kPa
Cell front displacement (μm)	14.66	14.59	11.98
Nucleus front displacement (μm)	6.12	5.44	2.93

**Fig. 8:**
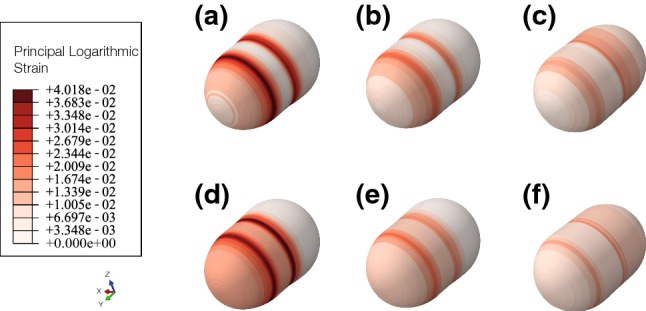
Absolute principal logarithmic strains in the nucleus. On top, absolute maximum strain, at the bottom, absolute minimum strain **a** maximum (4 kPa), **b** maximum, (8 kPa, control case), **c** maximum (16 kPa), **d** minimum (4 kPa), **e** minimum (8 kPa, control case), **f** minimum (16 kPa). Strains are higher when the nucleus has a Young modulus of 4 kPa

Regarding principal strains in the nucleus (Fig. 8), we can see how the less stiff nucleus is deforming pretty much than the control one. The lowest deformations are located in the rear of the nucleus in the three cases. The front part presents the highest deformations, due to actin depolymerization, and it generates higher stresses, whereas the rear part of the nucleus is compressing. The strains are bigger when the stiffness of the nucleus is 4 kPa. The strains are higher when the nucleus stiffness is lower.

Finally, the influence of the friction coefficient between the channel walls and the cell is analysed. The total displacement of the cell front after 160 min of simulation (Table 2) indicates that cell displacement is larger in those channels with higher friction coefficient.

**Table 2:** Cell displacement depending on the friction coefficient between the channel walls and the cell. The cell displaces further when the friction coefficient is higher

Friction coefficient	Cell displacement (μm)
0.4	14.16
0.5 (control case)	14.59
0.6	15.90

## Discussion and conclusions

In this work, we develop and implement an actin-based model to describe the individual cell migration process under confined conditions. In order to validate this model, we compare our results with in vitro experiments. The predicted velocity of the in silico cell is 0.1 μm/min which is of the same order to the values obtained in the literature (Liu et al. 2015; Irimia and Toner 2009). Moreover, the cell adopts the characteristic shape of confined migration, forming a tail in the back part of the cell, which can be observed in many in vitro experiments (Wilson et al. 2013; Irimia et al. 2007).

Except initial cell protrusion formation, all other steps in cell migration cycle involve dynamic interactions between the CSK and the nucleus. In mesenchymal migration, which is believed to drive cancer cell migration, the nucleus moves towards the cell rear (Calero-Cuenca et al. 2018). In contrast, in amoeboid migration, the nucleus moves towards the leading edge (Friedl et al. 2011). Nevertheless, it is unclear why the cell chooses between both types of migration. In our simulations, the nucleus moves first towards the rear part of the cell, corresponding to mesenchymal cell migration type.

After this movement, the leading edge is anchored to the substrate, resulting in forward pushing the nucleus (Cramer 2010). Our model predicts how the nucleus is placed closer to the rear of the cell in the first steps, and after that, it migrates within the whole cell.

In addition, we assume the cell as a solid and the model is implemented using finite elements, so we can study the mechanical behaviour of the cell. This is really important in processes as mechanotransduction, when the cell behaviour has a strong dependence on the forces exerted by the cell, and also on how the cell senses the environment. Other previous models assume the cell as a viscous fluid (Chen et al. 2018; Moure and Gomez 2018); thus, it is not possible to determine the stresses and strains inside the cell.

We perform several analyses in order to study the influence of different factors as the nucleus size and stiffness and the influence of the friction coefficient on the cell migration velocity.

The nucleus plays a key role in most of the cellular processes; in fact, its deformation is related to different cell processes such as differentiation, proliferation and also the way migration takes place (Vaziri et al. 2006; Friedl et al. 2011). Thus, we study the mechanical state of the cell nucleus during cell migration. The nucleus is the largest organelle in the cell, and it is larger than many pores encountered during migration in physiological tissues; in fact, it is the limiting structure in cell migration. In addition, experimental data reveal that the nucleus is almost ten times stiffer than the surrounding cytoplasm (Friedl et al. 2011). This combination of large size and relative rigidity of nucleus leads to the hypothesis that nucleus can impact the cells ability to migrate. The results show that the size of the nucleus has a great impact on the cell displacement, and the cell moves further when the nucleus is smaller. This result is in harmony to the ones obtained in Lautscham et al. (2015), who pointed out that smaller than average nucleus shows a considerably higher migration velocity. This could be due to the fact that the interstitial space is smaller than the nucleus, so the nucleus has to undergo high deformations in order to migrate. As the nucleus is the stiffer part of the cell, this deformation will became into a challenge to overcome. Work on tumour cells migrating through microfluidic platforms supports the hypothesis that substantial nuclear deformation results in reduced migration speeds (McGregor et al. 2016). So, migration is faster when the deformation of the cell nucleus is lower as predicted in the simulation.

In addition, when we study the influence of the nucleus stiffness, we obtain that the cell displacement is lower when the nucleus is stiffer. The stiffest nucleus is the one which suffers less deformations (Fig. 8). These results are in agreement with the results of McGregor et al. (2016). Lautscham et al. (2015) also pointed out that stiffer nucleus presents a greater resistance to migrate through confined spaces.

It is unclear if cells need to adhere to the substrate in order to properly migrate. Some studies indicate that in 3D confinement, migration can be achieved without specific adhesions (Lämmermann et al. 2008; Friedl 2010; Parsons et al. 2010). They stand for friction alone generating sufficient force to mediate cell body translocation. Bergert et al. (2015) directly measured friction coefficients on single cells using a microfluidic chip. They conclude that a threshold friction is required for cell motion, and that the cell velocity was higher with larger friction coefficients. In our simulation, the cell with the maximum friction coefficient is the one which displaces further. These results are consistent with in vitro cell migration experiments (Bergert et al. 2015). The cell can exert higher forces towards the channel wall, and it makes stronger adhesions. These factors allow the cell to move faster. Nevertheless, other authors suggest (Byun et al. 2013) that reduced friction may be a factor in enabling cancer cells to efficiently squeeze through tight spaces.

To develop these simulations of cell migration, several simplifications are necessary. First, the model does not take into account the membrane behaviour. However, as far as we know, it is still not clear if it plays an important role in cell migration from a mechanical point of view. Second, the friction coefficient between the cell and the channel wall is assumed constant; nevertheless, some works point out a variable friction coefficient in the channel wall (Liu and Gao 2015). Third, myosin contraction is assumed constant over time, although some research groups (Olsen et al. 1998) point out that myosin contraction depends on the stresses that the nucleus sense via mechanotransduction. This could be incorporated to the model making myosin dependent on the cell stress. In any case, these simplifications do not affect to the main conclusions obtained in this work.

In fact, the main conclusion in our work is that cell migration is the result of the complex action of different forces exerted by the interaction between F-actin, G-actin and myosin. These forces must act together to result in cell migration. In contrast with other studies (Recho et al. 2013) which support that cell motility is mainly based on myosin-induced contraction and does not require actin polymerization, we find that it is the cooperation between myosin contractility forces and actin processes (polymerization and depolymerization) which induce confined cell migration. Nevertheless, Lautscham et al. (2015) maintain that high contractile forces are necessary but not sufficient for invasion. According to Wilson et al. (2013), it can be seen that adherent F-actin plays a key role in cell migration too. It polymerizes towards the cell–channel interface, so the contact between them is always high, in order to allow cell migration. Therefore, in our work, we also conclude that cell adhesion to the substrate is crucial to achieve cell migration, and that in 3D cell motility it is important the existence of a high contact force.

In conclusion, in this work we propose a model which predict cell migration in confined spaces, simulating actin and myosin behaviour. The results confirm that myosin and actin have to act together to induce cell migration. Actin polymerization results in a displacement at the leading edge, myosin forces provoke a contraction at the rear part of the cell, and actin depolymerization is responsible of pulling the nucleus towards the front.
